# Leveraging data and policy interventions to strengthen tobacco control in the Eastern Mediterranean Region

**DOI:** 10.3389/fpubh.2025.1653026

**Published:** 2025-09-17

**Authors:** Randa K. Saad, Nayera S. Mostafa, Mahmoud Nabulsi, Sarah Al-Saket, Maysaa Nemer, Nour Obeidat, Yousef Khader

**Affiliations:** ^1^Global Health Development| Eastern Mediterranean Public Health Network, Amman, Jordan; ^2^Cairo Association against Smoking, Tuberculosis and Lung diseases (CASTLE), Cairo, Egypt; ^3^Community, Environmental, and Occupational Medicine Department, Ain Shams University, Cairo, Egypt; ^4^Royal Health Awareness Society, Amman, Jordan; ^5^Institute of Community and Public Health, Birzeit University, Ramallah, Palestine; ^6^Cancer Control Office, King Hussein Cancer Center, Amman, Jordan; ^7^Department of Public Health, Jordan University of Science and Technology, Irbid, Jordan

**Keywords:** Eastern Mediterranean Region, tobacco contro, MPOWER, policy, data

## Abstract

The “*Utilizing Tobacco Control Evidence to Inform Policies and Programs*” workshop focused on supporting Eastern Mediterranean Region (EMR) countries facing tobacco control challenges and seeking evidence-based mechanisms within the WHO MPOWER framework to strengthen tobacco control efforts. Participants from Egypt, Iraq, Jordan, Lebanon, and Palestine reviewed their tobacco control policies, identified barriers, and proposed countermeasures. Key challenges included weak enforcement of smoke-free laws and frequent violations due to an insufficient task force to monitor and ensure compliance with regulations. Even when violations are detected, penalties are low and often appealed. Inadequate data collection hinders evidence-based interventions and the ability to track the spread and emergence of tobacco and vaping products, such as waterpipes and, more recently, electronic cigarettes and heat-not-burn tobacco products. Tobacco industry interference further weakens political will and hampers policy enforcement. The rise of novel tobacco products adds regulatory complexities to laws with pre-existing loopholes. Workshop participants recommended increasing tobacco taxes in Iraq and Egypt to reduce tobacco accessibility. Expanding cessation services in public health facilities, particularly in Jordan and Palestine, was also recommended, along with public awareness campaigns to promote quitting. Improving tobacco surveillance, especially in Iraq, through tools like the Global Tobacco Surveillance System, was highlighted to enhance data collection and inform policies. Strengthening enforcement mechanisms, increasing fines, and improving coordination between public health and law enforcement were identified as critical measures for improving smoke-free policy compliance.

## 1 Introduction

Tobacco use is the single greatest preventable cause of death globally, killing up to half the people who use it (1). Tobacco control strategies are framed by the 2005 World Health Organization (WHO) Framework Convention on Tobacco Control (FCTC), a landmark public health treaty that was ratified by 182 Parties, representing 90% of the global population. The FCTC assists countries in mobilizing political and financial resources to implement effective tobacco control policies (2). Furthermore, Goal 3 of the United Nations Sustainable Development Goals (SDGs), adopted in 2015, commits to reducing premature mortality from non-communicable diseases (NCDs) by 30% by 2030 (Target 3.4). One of the key strategies for achieving this is strengthening the implementation of the WHO FCTC (Target 3.a) (3).

Ratification of the FCTC provides governments with an opportunity to comply with treaty obligations by implementing tobacco control measures, such as smoke-free policies, packaging and labeling regulations, marketing bans, cessation programs, sales restrictions to minors, and higher tobacco excise taxes. To support countries in implementing these demand-reduction measures, WHO introduced the MPOWER package, which outlines six evidence-based policies for reducing tobacco use (4). The MPOWER policies include monitoring tobacco use, protecting people from tobacco smoke, offering help to quit, warning about the dangers of tobacco, enforcing bans on tobacco advertising, and raising taxes on tobacco products.

While these policies are critical, many developing countries struggle to utilize data effectively or implement proven strategies for controlling the tobacco epidemic. This paper outlines insights gained from a workshop during the Eastern Mediterranean Public Health Network (EMPHNET) 8th Biennial Regional Conference, which aimed to assist countries in prioritizing action areas from the MPOWER package, including policies, programs, and data application, that have the biggest impact on addressing tobacco use prevalence, as well as the most political opportunity for action now in their countries.

Unlike previous regional tobacco control reports, which largely document policy status, this workshop generated *country-specific, consensus-based countermeasures* linked to MPOWER priorities. The participatory use of structured analysis tools (e.g., fishbone diagrams, prioritization exercises) produced actionable insights tailored to each country's political and cultural context.

## 2 Workshop description

The four-hour workshop aimed to support participating Eastern Mediterranean Region (EMR) countries in selecting a high-priority tobacco control action area (program or policy) where the use of data could be enhanced to inform and strengthen tobacco control measures. Its key objective was to help countries identify barriers to the effective use of data and exchange experiences regarding practical countermeasures to address these challenges. The workshop encouraged discussions around feasible, evidence-based decision-making for tobacco control in areas where both impact and political opportunity are greatest. As an example of successful data utilization, the United Against Tobacco and COVID (UATC) campaign was highlighted, providing participants with a real-world model of how data can guide both the strategic focus and the implementation of tobacco control programs.

The workshop was conducted in English and moderated by Mr. Mahmoud Nabulsi, Deputy Director General at the Royal Health Awareness Society in Jordan. It began with presentations from experts in tobacco control. Dr. Fatima Al-Awa, Regional Advisor for the Tobacco-Free Initiative at WHO EMRO, highlighted the status of tobacco control in the EMR and presented “quick wins” for implementing the FCTC and MPOWER framework. Dr. Randa Saad, Senior Technical Specialist at EMPHNET, followed with a presentation on the UATC campaign, while Dr. Indu B. Ahluwalia, Chief of CDC's Global Tobacco Control Branch, provided an overview of the Global Tobacco Surveillance System (GTSS) and its role in shaping global tobacco control policies.

Workshop participants were purposively selected through invitations extended to tobacco control focal points at ministries of health, civil society organizations, academic institutions, and international partners active in tobacco control across the Eastern Mediterranean Region. Thirty participants from Egypt, Iraq, Jordan, Lebanon, and Palestine attended.

A structured group exercise was used to guide the analysis (Table 1). Each country team reviewed its existing tobacco control policies and identified priority action areas. Participants then used a fishbone diagram to analyze root causes of barriers and collectively brainstorm countermeasures. Country groups presented their findings in plenary, where facilitators summarized key points and guided discussion.

**Table 1:** Summary of activities conducted during the workshop on using tobacco control evidence for policy and programs.

**Step**	**Description**
1. Identify tobacco control action areas	Participants reviewed current tobacco control policies and programs, assessing how well data were used in decision-making.
2. Prioritize action areas	Each country prioritized an action area for improvement, focusing on areas with high potential impact.
3. Identify barriers	Participants identified the most critical barriers to effective use of tobacco control data, ranking them by importance.
4. Analyze root causes	Using a fishbone diagram, participants analyzed the root causes of these barriers, exploring underlying factors.
5. Identify countermeasures	Practical methods to address the barriers were brainstormed, with attention to their effectiveness and feasibility.

Consensus was reached through iterative dialogue and group ranking: teams first agreed internally on priority barriers and countermeasures, and then the plenary validated these through discussion and verbal agreement. Detailed notes were taken by rapporteurs from EMPHNET, which were later synthesized thematically to inform this manuscript.

## 3 Findings

### 3.1 The current landscape of tobacco control in the Eastern Mediterranean Region

The first presentation outlined the alignment of tobacco control efforts in the EMR with global health objectives, including the SDGs and FCTC, emphasizing tobacco's role in the region's high burden of NCDs. Currently, 18% of adults in the region use tobacco. The presentation also discussed trends in tobacco use among youth and adults, noting mixed progress in reducing consumption.

Key challenges to the implementation of the MPOWER framework in the EMR include weak data collection, low prioritization of tobacco control policies, and poor implementation of smoke-free environments and cessation services. Emerging threats, such as novel tobacco products, inadequate enforcement of tobacco taxation, tobacco industry interference, ongoing conflicts, and disruptions caused by the COVID-19 pandemic, were also mentioned. Additionally, the discussion noted the effectiveness of tobacco taxation, while pointing out that many countries lag in enforcement and efforts to combat illicit trade. Many countries in the EMR still face significant and interrelated barriers to implementing effective tobacco control policies (Table 2).

**Table 2:** Key barriers and strategies to advancing tobacco control policies and programs.

**Barrier/ countermeasure**	**Description**
**Barrier**	
Political will and competing interests	Tobacco control, with its cost-effective measures in the long run, is often deprioritized in favor of other short-term health or economic issues, particularly in countries where the tobacco industry plays a significant economic role.
Framing of tobacco revenue as a necessary source of government revenue	This perspective, particularly among financial entities in governments, has contributed to slow (if any) support of proposed tobacco control measures.
Tobacco industry interference	The tobacco industry actively lobbies against tobacco control measures, particularly in low- and middle-income countries, weakening policy adoption and enforcement.
	“*Tobacco industry interference is one of the biggest challenges we face. Strengthening regulatory frameworks and increasing political resolve is essential to counter this influence*.”—Dr. Fatima Al-Awa.
Weak enforcement	Even where strong policies exist, enforcement is often lacking due to resource constraints, institutional limitations, or political corruption.
Insufficient data collection	Many countries lack reliable, up-to-date data on tobacco use and control measures, making it difficult to tailor interventions effectively.
**Countermeasure**	
Political advocacy	Presenting the economic benefits of tobacco taxation and the health savings from reducing tobacco-related diseases to build political will.
Multi-sectoral collaboration	Strengthening coordination between health, education, and enforcement sectors to build a unified voice for tobacco control.
Public campaigns	Personalizing the dangers of tobacco through emotional narratives to increase public support for stricter policies.
Capacity building	Enhancing data collection and analysis to ensure evidence is used effectively in policy-making.

Countries like Saudi Arabia and Oman were praised for their robust tobacco control policies, and their move toward plain packaging as well as raising tobacco prices.

“*While the EMR has seen some progress, many countries still lag in terms of enforcing smoke-free policies and providing accessible cessation services. Countries like Saudi Arabia and Oman have shown what is possible, but the gap remains large in the region*.”—Dr. Al-Awa.

### 3.2 The role of data in shaping tobacco control policies

Data emerged as a central theme, with discussions highlighting its critical role in formulating and refining tobacco control policies. The GTSS, which includes the Global Youth Tobacco Survey (GYTS) and Global Adult Tobacco Survey (GATS), is a series of globally standardized surveys designed to monitor tobacco use among youth and adults, along with key tobacco control indicators. The system is closely aligned with the FCTC and MPOWER measures, aiming to reduce tobacco demand globally.

Since its inception, the GTSS has expanded significantly. GYTS has surveyed approximately two million students across 188 countries since 1999, while GATS has been implemented in 36 countries, covering over 70% of the global adult population. This data has informed tobacco control policies and enabled countries to track the impact of their programs.

Data from GTSS informs tobacco control strategies and allows countries to track the effectiveness of interventions. However, many countries in the EMR are not fully utilizing the available data, often due to insufficient national infrastructure for data collection and limited capacity to analyze and apply the findings.

The discussion underscored the importance of integrating tobacco surveillance programs into national, regional, and global health systems to ensure data comparability and support evidence-based tobacco control policies.

“*Data is the backbone of our efforts—without real-time, robust data, we can't tailor our interventions to the specific needs of countries. Monitoring tobacco use through systems like GATS and GYTS is critical for shaping effective policies*.”—Dr. Ahluwalia.

### 3.3 Success stories in tobacco control

The UATC campaign was launched in response to research linking tobacco use with severe COVID-19 outcomes. In 2022, 17 EMR countries temporarily banned waterpipe use in public spaces to reduce virus transmission, marking a shift toward stronger tobacco control measures.

The FCTC Article 12 urges countries to warn people about the dangers of tobacco use (5), while the MPOWER framework underscores the role of media in promoting tobacco control policies, maintaining tobacco control on social and political agendas, and shaping public and policymaker views (4). To this end, the UATC campaign was launched in Egypt, Iraq, Jordan, and Palestine in 2022 as a collaborative effort between the US CDC, EMPHNET, and Vital Strategies (6). The campaign aimed to raise awareness about tobacco's harmful effects, promote cessation, and support smoke-free policies. Countries were selected due to their high smoking rates, secondhand smoke (SHS) exposure, shared language, and geographic proximity, which allowed for cross-pollination of campaign messages.

The campaign utilized various media platforms to reach diverse audiences. Culturally relevant, evidence-based messaging adapted from a Turkish anti-tobacco initiative was central to the campaign. Emotional narratives featuring relatable stories were used to evoke fear, disgust, and sadness, proven to be effective in promoting smoking cessation and behavior change.

The GTSS's role during the COVID-19 pandemic was highlighted, where data from the system informed the UATC campaign. This campaign reached 50 million individuals across Egypt, Iraq, Jordan, and Palestine in 2022, raising awareness about the link between tobacco use and COVID-19 outcomes, while spurring governments to take action and increasing demand for cessation services (7). Post-campaign evaluations showed increased visits to cessation clinics, heightened public discourse on tobacco harms, and policy shifts like stricter smoke-free regulations. The campaign also strengthened government backing, particularly in Jordan, where discussions on tightening tobacco policies gained traction. Building on its success, the second phase of the campaign (2023–2024) focused its efforts on Jordan and Palestine to sustain progress (8).

“*In Jordan, public support for tobacco control measures increased significantly after the campaign, and demand for cessation services rose—a clear example of how effective evidence-based campaigns can be*.”—Dr. Saad.

### 3.4 Country-specific findings and countermeasures

#### 3.4.1 Iraq: addressing tobacco advertising, promotion, and sponsorship

Participants from Iraq identified the ban on tobacco advertising, promotion, and sponsorship as a priority area for using data to drive impactful interventions. The primary barrier is the lack of sufficient data to guide policy decisions. Limited financial and human resources for data collection and analysis hinder efforts to assess the scope of the problem or evaluate intervention outcomes.

Proposed countermeasures include partnering with international and local funders to support studies on the prevalence and impact of tobacco marketing. These efforts would generate evidence to inform policies and strengthen advocacy. Additionally, fostering a culture of evidence-based decision-making within relevant institutions would improve the design and implementation of tobacco control measures.

Participants also emphasized the importance of introducing or enhancing legal frameworks to govern, facilitate, and mandate data collection and sharing on tobacco marketing, ensuring that policymakers and health officials have access to accurate and timely information. Strengthening these mechanisms would improve transparency and accountability, making it easier to enforce advertising bans and measure their impact. By addressing the financial, cultural, and legislative barriers to data collection, Iraq can make significant progress in using data to combat tobacco advertising and reduce smoking rates.

#### 3.4.2 Lebanon, Palestine, and Jordan: protecting against secondhand smoke

Participants from Lebanon, Palestine, and Jordan identified SHS protection as a priority area for improving data use in tobacco control.

In Lebanon, a major barrier is the perception of tobacco use as a profitable industry in a country that relies on tourism, as well as tobacco cultivation and manufacturing. Moreover, waterpipe tobacco use is deeply embedded in the culture, passed down through generations, making it challenging to address. Suggested countermeasures included raising public awareness about the health risks of SHS through targeted, data-driven campaigns and using evidence to advocate for stricter regulations. Engaging key stakeholders, such as policymakers, health authorities, and the tourism sector, is essential for promoting alternative economic opportunities that reduce reliance on the tobacco industry. Community engagement and educational efforts to reduce the cultural normalization of waterpipe tobacco use are also critical for shifting societal attitudes over time.

In Palestine, weak enforcement of smoking ban laws remains a significant barrier to addressing SHS exposure. The issue is compounded by political instability, which diverts attention and resources toward conflict-related concerns, reducing the focus on public health regulations. Cultural norms, particularly the acceptance of waterpipe smoking, further complicate efforts, as smoking is deeply ingrained in family and social life. Economic pressures also play a role, with many businesses, such as cafes and restaurants, allowing smoking to attract customers, especially in the context of high unemployment. There is also a lack of public awareness about the dangers of SHS, and public health campaigns have not adequately highlighted its risks, particularly in private spaces like homes and cars. Furthermore, limited data on SHS exposure and insufficient resources for enforcement exacerbate the issue.

To address these challenges, participants proposed several countermeasures. First, targeted public awareness campaigns are needed to educate the population on the risks of SHS, especially in private settings. Engaging community leaders and influencers to challenge the cultural normalization of smoking, particularly waterpipe use, is key to changing social attitudes. Strengthening collaboration with international and local organizations could help secure the necessary funding and resources for data collection and public health initiatives, which are vital for informing evidence-based policies and interventions.

Exploring economic alternatives for businesses that rely on allowing smoking could reduce economic disruptions. Incentives for creating smoke-free environments in cafes and restaurants could encourage compliance while preserving business interests. Finally, improving law enforcement capacity through training, better monitoring, and stronger penalties for violations, along with fostering multi-sectoral collaboration, would enhance the enforcement of smoking bans.

In Jordan, a key priority for reducing SHS exposure is increasing public awareness and ensuring the effective enforcement of tobacco control laws. The primary barrier is the lack of authority and capacity among relevant institutions to consistently enforce existing laws, undermining tobacco control efforts. Several underlying causes contribute to this, including the absence of regular monitoring systems, low fines for violations, and poor coordination between public health agencies, law enforcement, and local governments. Without consistent monitoring, violations often go unpunished, and enforcement becomes sporadic and ineffective. Additionally, the low penalties provide little deterrence for individuals or businesses that ignore smoking bans.

Lack of coordination between authorities further fragments tobacco control efforts. Without a unified strategy and collaborative approach, enforcement efforts lose impact. There is also a significant gap in public awareness programs aimed at educating the population about the risks of tobacco use and the importance of following smoking bans. Many people remain unaware of the dangers of SHS or the legal consequences of violating tobacco control laws.

Jordan should establish a more robust and consistent monitoring and enforcement system, with regular inspections and stronger penalties for violations, to significantly enhance compliance. Increasing fines for non-compliance could also serve as a stronger deterrent. Improved coordination between public health authorities, law enforcement, and other stakeholders is essential for developing a comprehensive and unified strategy to combat tobacco use. Finally, launching sustained public awareness campaigns would educate the public about the health risks of SHS and emphasize the importance of adhering to tobacco control laws. Together, these actions would help Jordan strengthen its tobacco control efforts and protect public health.

#### 3.4.3 Egypt: education and communication in tobacco control

Participants from Egypt identified education and communication as the top priority for strengthening tobacco control efforts. This includes raising awareness about the health risks of tobacco use and promoting behavior change through effective communication strategies. The primary barrier to achieving these goals is the limited use of local, country-specific data to inform and guide public health initiatives. This is driven by a lack of resources for data collection and analysis, insufficient political support for prioritizing tobacco control, and poor coordination between key stakeholders, including the Ministry of Health and Population (MoHP), other government agencies, and non-governmental organizations (NGOs).

Without adequate resources, conducting the research needed to develop evidence-based education and communication strategies becomes difficult. Moreover, the lack of political support means tobacco control efforts are often deprioritized, leading to limited funding and weak advocacy efforts. This political landscape exacerbates the challenge of enforcing existing tobacco control laws and developing new initiatives. Additionally, although the Egyptian community witnessed several tobacco control initiatives during the last decade, the absence of strong coordination between official bodies and NGOs creates fragmentation.

To address these challenges, participants suggested increasing the number of data-driven public awareness campaigns focused on the dangers of tobacco use and the benefits of quitting and tailoring these campaigns to local contexts. Establishing Memoranda of Understanding (MoUs) between NGOs and the MoHP could strengthen partnerships and improve coordination. NGOs, with their flexibility and community outreach expertise, could complement government efforts by reaching underserved populations. Finally, engaging the private sector for sponsorships could provide much-needed funding and resources for education and communication efforts. Through corporate social responsibility (CSR) programs, private companies could support tobacco control campaigns, research, and capacity-building activities.

### 3.5 Practical countermeasures for improving policy implementation

During group discussions, participants identified several practical countermeasures to overcome the barriers in their respective countries, listed in Table 2.

## 4 Discussion

The workshop highlighted several themes that are critical for advancing tobacco control efforts in the EMR. While there has been progress, the region continues to face significant challenges in the implementation and enforcement of tobacco control policies, particularly in countries with strong tobacco industry influence and limited political will. To overcome these, strengthening of tobacco control policies, increasing taxation, expanding cessation services, and curbing tobacco industry influence are necessary.

One of the most significant barriers to effective tobacco control identified in this workshop is tobacco industry interference, which is consistent with findings from other regions (9). The tobacco industry employs tactics that undermine public health efforts, such as lobbying, litigation, and promoting misleading narratives about the economic benefits of tobacco cultivation and sales (10, 11). These strategies are particularly pervasive in low- and middle-income countries, where regulatory frameworks are weaker, and enforcement mechanisms are often underfunded (10). Workshop participants acknowledged that strengthening political resolve and ensuring robust regulatory frameworks to combat tobacco industry influence is essential for progress in tobacco control, a point that has been echoed in global tobacco control literature (12, 13).

Another recurring issue across EMR countries is the lack of sufficient data collection and utilization for shaping tobacco control policies. The discussion on GTSS underscored the importance of standardized data in tracking tobacco use trends and assessing the impact of policies. However, many countries in the region are not fully leveraging available data due to inadequate infrastructure and capacity for data analysis. Previous studies have emphasized the critical role of real-time, high-quality data in developing evidence-based interventions and improving policy implementation (14–16). Integrating tobacco surveillance into national health systems is vital for ensuring data-driven decision-making (17), and this workshop highlighted the need for capacity building in data analysis to make full use of systems like GTSS (18).

The success of the UATC campaign offers an exemplary case of how data-driven, culturally tailored public awareness campaigns can catalyze policy change and behavior modification, particularly when combined with strong monitoring and evaluation frameworks (21, 22). The campaign's use of emotional narratives and culturally relevant messages was particularly effective, a strategy supported by behavioral research showing that emotionally evocative health warnings are more likely to influence smoking behavior than purely informational campaigns (19, 20).

Lastly, the workshop emphasized the importance of multi-sectoral collaboration and political advocacy to build momentum for stronger tobacco control policies. Engaging key stakeholders from the health, education, and law enforcement sectors, as well as leveraging CSR programs in the private sector, can help overcome resource constraints and build public support for tobacco control measures. Previous research has highlighted the need for cross-sector partnerships to create sustainable policy changes, especially in resource-constrained settings (23–25). The proposed MoUs between NGOs and ministries of health underscore the need for coordinated action and shared responsibility in tackling the tobacco epidemic.

Beyond describing barriers, this analysis highlights *why* they persist in the region. Weak enforcement of smoke-free laws reflects chronic underfunding, competing political priorities, and fragmented authority between ministries and law enforcement. Tobacco industry interference persists because of strong lobbying, economic reliance on tobacco revenues, and weak implementation of FCTC Article 5.3 protections. Cultural acceptance of waterpipe smoking, particularly in social and tourism contexts, sustains demand and makes enforcement politically sensitive. These dynamics explain the gap between formal policies and actual implementation.

Countermeasures identified during the workshop, such as increasing penalties, strengthening multi-sectoral enforcement, and promoting alternative livelihoods for businesses reliant on smoking, are feasible but require political will and inter-ministerial coordination. Embedding cessation services within primary healthcare platforms is particularly realistic, given existing infrastructure and alignment with WHO Best Buys.

To advance tobacco control in the Eastern Mediterranean Region, workshop participants proposed the following actionable measures:

- Align excise taxes with WHO's recommended benchmark of at least 75% of the retail price; Egypt and Iraq were highlighted as priority countries.
- Establish independent compliance units or inter-ministerial task forces to conduct regular inspections, supported by increased fines that serve as real deterrents.
- Expand access by integrating brief advice into primary health care, ensuring national quitlines are functional, and making nicotine replacement therapy available.
- Institutionalize participation in the Global Tobacco Surveillance System every 3–5 years and allocate domestic resources to sustain national surveys.
- Launch sustained, culturally tailored mass media campaigns, modeled on the United Against Tobacco and COVID campaign, with proven reach and effectiveness.

The findings should be interpreted in light of several limitations. First, the workshop was limited in duration (4 h) and scope (five countries), which restricted the breadth of perspectives captured. Participants were primarily public health and civil society representatives, which may have biased discussions toward health-sector solutions rather than broader economic or political considerations. Second, the consensus-based process may have amplified dominant voices, while minority views may not have been fully represented. Finally, as findings were generated through a single workshop without follow-up validation, they should be viewed as preliminary insights requiring further stakeholder engagement.

This workshop adds novelty to regional tobacco control efforts by moving beyond descriptive reporting to a participatory, consensus-driven process. Unlike prior regional tobacco control assessments, which primarily document policy status, this exercise applied structured analytical tools (e.g., fishbone diagrams, prioritization exercises) to co-develop actionable, country-specific countermeasures. This participatory methodology highlights the value of engaging policymakers, academics, and civil society jointly in shaping feasible strategies, and demonstrates how regional workshops can generate practical outputs aligned with MPOWER and FCTC commitments.

## 5 Conclusion

The findings reaffirm the critical role of evidence-based policies, political commitment, and cross-sector collaboration in advancing tobacco control in the EMR. By addressing barriers related to tobacco industry interference, improving data collection and utilization, and investing in public awareness campaigns, countries in the region can make meaningful progress in reducing tobacco use and its associated health burdens. Further research and continuous capacity building will be essential in ensuring long-term success in tobacco control.

## Data Availability

The original contributions presented in the study are included in the article/supplementary material, further inquiries can be directed to the corresponding author.
